# Clinical characteristics of 276 hospitalized patients with coronavirus disease 2019 in Zengdu District, Hubei Province: a single-center descriptive study

**DOI:** 10.1186/s12879-020-05252-8

**Published:** 2020-07-29

**Authors:** Yiping Wei, Weibiao Zeng, Xiangyun Huang, Junyu Li, Xingting Qiu, Huadong Li, Dinghua Liu, Zhaofeng He, Wenzhong Yao, Ping Huang, Chao Li, Min Zhu, Chunlan Zhong, Xingen Zhu, Jiansheng Liu

**Affiliations:** 1 Department of Thoracic Surgery, The Second Affiliated Hospital of Nanchang University, Nanchang, China; 2 Suizhou Zengdu Hospital, Suizhou, China; 3 Department of Radiotherapy, Jiangxi Cancer Hospital, NanChang, China; 4 Department of CT&MRI, The First Affiliated Hospital of Gannan Medical University, Ganzhou, China; 5 Department of Respiratory Medicine, Chongyi County People’s Hospital, Ganzhou, China; 6 Department of Critical Medicine, Ganzhou Tumor Hospital, Ganzhou, China; 7 Department of Critical Care Medicine, Dingnan People’s Hospital, Dingnan, China; 8 Department of Critical Medicine, Anyuan People’s Hospital, Ganzhou, China; 9 Department of Infectious Disease, Xingguo People’s Hospital, Ganzhou, China; 10 Department of Respiratory Medicine, Jiangxi Province Hospital of Integrated Chinese and Western Medicine, Nanchang, China; 11 Department of General Practice, The First People’s Hospital of Fuzhou, Fuzhou, China; 12 Department of Pediatric Neurology, Ganzhou Women’s and Children’s Hospital of Jiangxi Province, Ganzhou, China; 13 Neurosurgery Department, The Second Affiliated Hospital of Nanchang University, Nanchang, China; 14 Department of Respiratory Medicine, Ganzhou People’s Hospital, No.17 Hongqi Avenue, Ganzhou City, 341000 Jiangxi Province China

**Keywords:** COVID-19, SARS-CoV-2, Echocardiography, Clinical characteristics

## Abstract

**Background:**

We aimed to report the epidemiological and clinical characteristics of hospitalized patients with coronavirus disease-19 (COVID-19) in Zengdu District, Hubei Province, China.

**Methods:**

Clinical data on COVID-19 inpatients in Zengdu Hospital from January 27 to March 11, 2020 were collected; this is a community hospital in an area surrounding Wuhan and supported by volunteer doctors. All hospitalized patients with COVID-19 were included in this study. The epidemiological findings, clinical features, laboratory findings, radiologic manifestations, and clinical outcomes of these patients were analyzed. The patients were followed up for clinical outcomes until March 22, 2020. Severe COVID-19 cases include severe and critical cases diagnosed according to the seventh edition of China’s COVID-19 diagnostic guidelines. Severe and critical COVID-19 cases were diagnosed according to the seventh edition of China’s COVID-19 diagnostic guidelines.

**Results:**

All hospitalized COVID-19 patients, 276 (median age: 51.0 years), were enrolled, including 262 non-severe and 14 severe patients. The proportion of patients aged over 60 years was higher in the severe group (78.6%) than in the non-severe group (18.7%, *p* < 0.01). Approximately a quarter of the patients (24.6%) had at least one comorbidity, such as hypertension, diabetes, or cancer, and the proportion of patients with comorbidities was higher in the severe group (85.7%) than in the non-severe group (21.4%, *p* < 0.01). Common symptoms included fever (82.2% [227/276]) and cough (78.0% [218/276]). 38.4% (106/276) of the patients had a fever at the time of admission. Most patients (94.9% [204/276]) were cured and discharged; 3.6% (10/276) deteriorated to a critical condition and were transferred to another hospital. The median COVID-19 treatment duration and hospital stay were 14.0 and 18.0 days, respectively.

**Conclusions:**

Most of the COVID-19 patients in Zengdu had mild disease. Older patients with underlying diseases were at a higher risk of progression to severe disease. The length of hospital-stay and antiviral treatment duration for COVID-19 were slightly longer than those in Wuhan. This work will contribute toward an understanding of COVID-19 characteristics in the areas around the core COVID-19 outbreak region and serve as a reference for decision-making for epidemic prevention and control in similar areas.

## Background

In December 2019, a series of pneumonia cases with similar symptoms were reported in Wuhan, Hubei Province, China [1]. That pneumonia was later named coronavirus disease 2019 (COVID-19) by the World Health Organization (WHO) [2]. The causative pathogen was identified as a novel enveloped RNA beta coronavirus named severe acute respiratory syndrome coronavirus 2 (SARS-CoV-2) [3]. COVID-19 is highly contagious and spreads rapidly through human-to-human transmission [4–6]. As of March 29, 2020, there were 81,470 confirmed COVID-19 cases and 3304 deaths in China, including 67,801 confirmed cases and 3186 deaths in Hubei Province, and 721,584 confirmed cases and 33,958 deaths worldwide. However, many infected people have not been counted owing to a lack of timely diagnosis. COVID-19 is a global pandemic. Therefore, a comprehensive and in-depth understanding of the epidemiological and clinical characteristics of COVID-19 is imperative for controlling the pandemic as soon as possible.

The number of COVID-19 cases in Wuhan was large, the spread was fast, and the fatality rate was high. Most of the clinical characteristics of COVID-19 have been summarized from the samples of patients in Wuhan [7]. Controlling the epidemic in the areas around the core COVID-19 outbreak region is an important link in blocking the spread of the disease. The Chinese government has enlisted many volunteer doctors to support hospitals in these key areas. However, there are few reports on the clinical characteristics of COVID-19 inpatients in these areas [8, 9]. Thus, this study collected clinical data for COVID-19 inpatients in Zengdu Hospital, a community hospital supported by 95 volunteer doctors and nurses from Jiangxi Province (about 300 miles from Zengdu District). We describe the epidemiology, clinical features, laboratory findings, imaging features, and outcomes of COVID-19 inpatients in Zengdu District, which is a 3-h drive from Wuhan City. We hope that our work will contribute toward an understanding of COVID-19 characteristics in the areas around the core COVID-19 outbreak region and provide a decision-making reference for epidemic prevention and control in similar areas.

## Methods

### Data sources

The study was approved by the institutional ethics board of Suizhou Zengdu Hospital, which was established by the Chinese government to treat COVID-19 patients in Zengdu District. All the patients diagnosed with COVID-19, according to the interim guidance from the WHO [10], in Zengdu Hospital from January 27 to March 11 were admitted and included in this study. The patients were followed up for clinical outcomes until March 22, 2020. Only laboratory-confirmed cases that were defined as positive based on the results of high-throughput sequencing or real-time reverse-transcriptase–polymerase chain reaction (RT-PCR) assay of nasal and pharyngeal swab samples were included. These confirmatory assays for SARS-CoV-2 were performed at the Suizhou CDC in accordance with the guidelines developed by the WHO [11]. Medication and treatment measures were selected according to the scheme recommended in the guidelines and each patient’s condition [12].

A team of doctors who had treated these patients extracted the recent exposure history, clinical symptoms, laboratory findings, radiologic manifestations, and clinical outcomes from patients’ medical records. All patients underwent at least one chest computed tomography (CT) scan, and data were extracted after the scans were reviewed by a dedicated imaging physician. All laboratory tests were performed according to treatment needs. The researchers obtained the outcome data of transferred patients by contacting the hospitals that received these patients, and also contacted the patients by phone if anything was unclear or information necessary for the study was missing from the medical record.

### Study definitions

According to the national treatment guideline, COVID-19 severity was defined as mild, moderate, severe, or critical [13]. The mild type was defined as mild clinical symptoms and no radiological manifestations of pneumonia. The moderate type was defined as respiratory symptoms and pneumonia on imaging. The disease was defined as severe if one of the following criteria was met: respiratory rate of ≥30 beats per minute; finger oxygen saturation of ≤93% at resting state; and arterial blood oxygen partial pressure (PaO_2_)/oxygen concentration (FiO_2_) of ≤300 mmHg. The critical type was defined as respiratory failure or shock and requirement of mechanical ventilation or intensive care unit (ICU) monitoring and treatment. Accordingly, the patients were divided into a non-severe group (mild or moderate disease type) and severe group (severe or critical disease type). Due to limited medical facilities at the Zengdu Hhospital, critical patients were transferred to hospitals with superior treatment facilities. The incubation period was defined as the interval between the patient’s earliest date of exposure to the transmission source and the date of the initial symptom. For patients who had recently visited Wuhan, the earliest date of exposure was estimated as the median date of their stay in Wuhan; for patients who had been in contact with people returning from Wuhan, the earliest date of exposure was considered to be the earliest contact date, the earliest date of exposure was considered to be the earliest contact date. Fever was defined as an axillary temperature of ≥37.5 °C. Lymphopenia, eosinopenia, and thrombocytopenia were defined as lymphocyte, eosinophil, and platelet counts of less than 1500, 100, and 150,000 of the corresponding cells per cubic millimeter. The smoking index was equal to the product of the number of cigarettes per day and smoking years. The length of COVID-19 treatment was defined as the time interval from patient admission to the meeting of the cure and discharge criteria of the Chinese management guidelines for COVID-19 (version 6.0) [12]. The cure and discharge criteria were as follows: Normal body temperature for more than 3 days; significantly improved respiratory symptoms; significantly improved acute exudative lesions on pulmonary imaging; and two consecutive negative results of the nucleic acid tests of sputum, nasopharyngeal swabs, and other respiratory tract samples.

### Study outcomes

The primary composite end points were discharge from the hospital owing to being cured and transfer to another hospital because of condition deterioration. The secondary end points were cure or discharge rate and the length of hospital stay.

### Statistical analyses

Statistical analyses were performed with SPSS (v.18.0; SPSS Inc., Chicago, IL, USA). Continuous variables are described as median values and interquartile ranges (IQRs), and categorical variables are reported as numbers and percentages. We used the Mann-Whitney U test, χ^2^ test, or Fisher’s exact test to compare differences between the two groups. A two-sided α of less than 0.05 was considered statistically significant.

## Results

### Demographic and clinical characteristics

We obtained data on the demographic characteristics, symptoms, and outcomes for 276 patients hospitalized in Suizhou Zengdu Hhospital as of March 11, 2020. The severe group included 14 (5.1%) patients while the non-severe group included 262 (94.9%) patients. The demographic and clinical characteristics of the patients are shown in Table 1. Forty-three (15.6%) of the 276 patients had visited Wuhan within 14 days before the study enrollment; 60.1% (166/276) of the patients had come into contact with people who had travelled to Wuhan or were diagnosed with COVID-19. The remaining 67 patients reported they had not been to Wuhan, and it was unclear how these patients had been exposed to the transmission source; none of the patients had a history of exposure to the Huanan seafood wholesale market or a wild animal. The incubation period calculated based on the data from 71 patients with a known exposure time was 6 days (IQR, 4–7 days). The longest incubation period was 20 days. A nurse in the fever clinic of Suizhou Zengdu Hhospital was the only medical staff included in the study.

**Table 1:** Clinical characteristics of 276 patients with COVID-19 on admission

Characteristic	All Patients	Disease Severity^a^		*p* value
	(*N* = 276)	Non-severe (*N* = 262)	Severe (*N* = 14)	
Age				
Median (IQR) — yr	51.0 (41.0–58.0)	50.0 (39.0–57.0)	65.0 (60.0–72.8)	< 0.01
Distribution — no./total no. (%)				
0–19 yr	4/276 (1.4)	4/262 (1.5)	0	0.50
20–59 yr	212/276 (76.8)	209/262 (79.8)	3/14 (21.4)	< 0.01
> 60 yr	60/276 (21.7)	49/262 (18.7)	11/14 (78.6)	< 0.01
Male sex-no./total no. (%)	155/276 (56.2)	145/262 (55.3)	10/14 (71.4)	0.24
Smoking Index ^b^ — no./total no. (%)				0.57
0	192/220 (87.2)	182/208 (87.5)	10/12 (83.3)	
1–199	7/220 (3.2)	7/208 (3.4)	0/12 (0)	
200–399	6/220 (2.7)	5/208 (2.4)	1/12 (8.3)	
≥ 400	15/220 (6.8)	14/208 (6.7)	1/12 (8.3)	
Median BMI (IQR)	23.7 (22.0–25.4)	23.7 (21.8–25.4)	24.2 (22.5–25.5)	0.55
Exposure to source of transmission within past 14 days — no./total no. ^c^				
Recently visited Wuhan	43/276 (15.6)	42/262 (16.0)	1/14 (7.1)	0.37
Had contact with people who visited Wuhan or were diagnosed with COVID-19	166/276 (60.1)	157/262 (59.9)	9/14 (64.3)	0.75
Contact with wildlife	0	0	0	
Median incubation period (IQR) — days ^d^	6.0 (4.0–9.0)	6.0 (4.0–9.0)	6.0 (5.0–9.0)	0.54
Fever on admission				
Patients — no./total no. (%)	106/276 (38.4)	99/262 (37.8)	7/14 (50.0)	0.36
Median temperature (IQR)—°C	37.2 (36.6–37.9)	37.2 (36.6–37.9)	37.6 (36.6–38.1)	0.56
Distribution of temperature — no./total no. (%)				
< 37.5 °C	170/276 (61.6)	163/262 (62.2)	7/14 (50.0)	0.37
37.5–38.0 °C	54/276 (19.6)	51/262 (19.5)	3/14 (21.4)	0.86
38.1–39.0 °C	41/276 (14.9)	39/262 (14.9)	2/14 (14.3)	0.95
> 39.0 °C	11/276 (4.0)	9/262 (3.4)	2/14 (14.3)	0.04
Fever during hospitalization				
Patients — no./total no. (%)	207/276 (75.0)	195 /262 (74.4)	12/14 (85.7)	0.34
Median highest temperature (IQR)—°C	38.2 (37.5–38.9)	38.2 (37.4–38.9)	38.9 (8.3–39.6)	0.47
Distribution of temperature — no./total no. (%)				
< 37.5 °C	69/276 (25.0)	67/262 (25.6)	2/14 (14.3)	0.34
37.5–38.0 °C	57/276 (20.7)	56/262 (21.4)	1/14 (7.1)	0.20
38.1–39.0 °C	107/276 (38.8)	103/262 (39.3)	4/14 (28.6)	0.41
> 39.0 °C	43/276 (15.6)	36/262 (13.7)	7/14 (50.0)	< 0.01
Symptoms — no./total no. (%)				
Fever	227/276 (82.2)	219/262 (84.4)	8/14 (57.1)	0.01
Conjunctival congestion	2/276 (0.7)	2/262 (0.8)	0/14 (0)	0.90
Nasal congestion	8/276 (2.9)	8/262 (3.1)	0/14 (0)	0.66
Headache	24/276 (8.7)	22/262 (8.4)	2/14 (14.3)	0.78
Cough	218/276 (78.0)	204/262 (77.9)	14/14 (100.0)	0.02
Sore throat	26/276 (9.4)	23/262 (8.8)	3/14 (21.4)	0.27
Sputum production	137/276 (49.6)	127/262 (48.5)	10/14 (71.4)	0.16
Fatigue	141/276 (51.1)	133/262 (50.8)	8/14 (57.1)	0.64
Hemoptysis	1/276 (0.4)	0/262 (0)	1/14 (7.1)	0.06
Shortness of breath	42/276 (15.2)	36/262 (13.7)	6/14 (42.9)	< 0.01
Nausea or vomiting	23/276 (8.3)	20/262 (7.6)	3/14 (21.4)	0.10
Diarrhea	6/276 (2.2)	5/262 (1.9)	1/14 (7.1)	0.71
Myalgia or arthralgia	26/276 (9.4)	24/262 (9.2)	2/14 (14.3)	0.84
Comorbidities — no./total no. (%)				
Any	68/276 (24.6)	56/262 (21.4)	12/14 (85.7)	< 0.01
Hypertension	47/276 (17.0)	39/262 (14.9)	8/14 (57.1)	< 0.01
Chronic obstructive pulmonary disease	7/276 (2.5)	5/262 (1.93)	2/14 (14.3)	0.04
Diabetes	14/276 (5.1)	12/262 (4.6)	2/14 (14.3)	0.32
Coronary heart disease	12/276 (4.0)	8/262 (5.2)	4/14 (28.6)	< 0.01
Cerebrovascular disease	6/276 (2.2)	5/262 (2.0)	1/14 (7.1)	0.71
Cancer ^e^	3/276 (1.1)	2/262 (0.8)	1/14 (7.1)	0.36

*IQR* interquartile range; *BMI* body mass index; *Covid-19* coronavirus disease 2019

^a^ Severe group needs to meet one of the following criteria: respiratory rate ≥ 30 beats per minute; finger oxygen saturation ≤ 93% at resting state; arterial blood oxygen partial pressure (PaO_2_)/oxygen concentration (FiO_2_) ≤ 300 mmHg; has respiratory failure or shock; required mechanical ventilation or intensive care unit monitoring and treatment

^b^ Smoking index was equal to the product of the number of cigarettes per day and years of smoking

^c^ 95 patients were unsure if they had been exposed to a source of transmission

^d^ Incubation period for 205 people could not be determined

^e^ Included any kind of cancer

The median age of the patients was 51 years (IQR, 41–58 years). The patients in the severe group were significantly older than those in the non-severe patients (median age: 65 years [IQR, 60.0–72.8 years] vs 50 years [IQR, 39.0–57.0 years], *p* < 0.01). Male patients accounted for 56.2% of all patients. A history of smoking was noted for 12.8% of the 220 patients with smoking index data. The median body mass index (BMI) of all the patients included was 23.7 (IQR, 22.0–25.4). The most common symptom of COVID-19 was fever, which was observed in 82.2% (227/276) of the patients. The other common symptoms were cough (78.0%, 218/276), fatigue (51.1%, 141/276), sputum production (49.6%, 137/276), and shortness of breath (15.2%, 42/276). Fever at the time of admission was noted in 38.4% (42/276) of the patients, while fever during hospitalization was noted in 75.0% (207/276) of the patients. At least one comorbidity was reported in 24.6% (68/276) of the patients, with the most common comorbidity being hypertension (17.0%, 47/276). Most of the severe patients (85.7%, 12/14) had at least one comorbidity; this percentage was significantly higher than that among the non-severe patients (21.4%, 56/262).

### Radiologic and laboratory findings

**Fig. 1:**
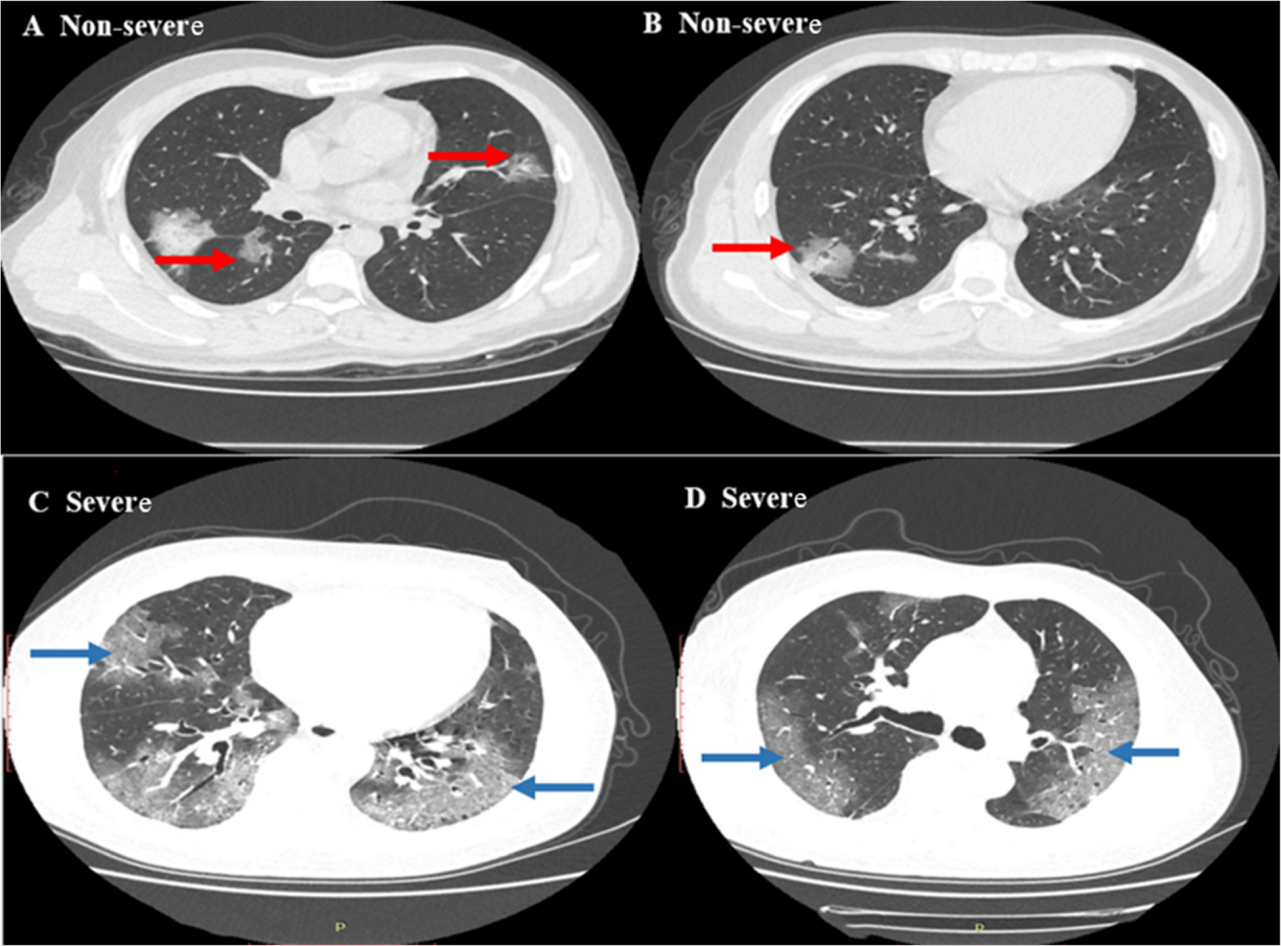
Typical chest computed tomography imaging of COVID-19 patients. Panels **a** and **b** show chest computed tomography images from a middle-aged male with non-severe COVID-19 at time of admission, showing several scattered ground-glass opacities (A, B the red arrow point). Panel **c** and **d** show chest computed tomography imaging findings from a middle-aged male with severe COVID-19 at time of admission, showing bilateral patchy shadows and consolidation (C, D the blue arrow point). COVID-19: coronavirus disease 2019

Table 2 shows the results of radiology and laboratory tests at admission. All 276 patients underwent CT at admission, and abnormal results were obtained for 95.7% (264/276) of the patients. The most common chest CT findings were bilateral patchy shadows (84.1%, 232/276) and ground-glass–like shadows (80.1%, 221/276). Figure 1 shows typical ground-glass shadows and bilateral patchy shadows in two patients.

**Table 2:** Radiographic and laboratory findings of 276 patients with COVID-19 on admission to hospital^a^

Variable	All Patients	Disease Severity		*p* value
	(N = 276)	Non-severe (N = 262)	Severe (N = 14)	
Abnormalities on chest CT — no./total no. (%)	264/276 (95.7%)	250/262 (95.4%)	14/14 (100.0%)	0.53
Ground-glass opacity	221/276 (80.1%)	209/262 (79.8%)	12/14 (85.7%)	0.45
Local patchy shadowing	17/276 (6.4%)	17/262 (6.5%)	0	0.40
Bilateral patchy shadowing	232/276 (84.1%)	220/262 (84.0%)	12/14 (85.7%)	0.41
Interstitial abnormalities	40/276 (14.5%)	37/262 (14.1%)	3/14 (21.4%)	0.54
Laboratory findings				
White-cell count				
Median (IQR) — per mm^3^	4700 (3800–6100)	4700 (3800–6000)	6000 (4000–7100)	0.27
Distribution — no./total no. (%)				
> 10,000 per mm^3^	16/276 (5.8)	15/262 (5.7)	1/14 (7.1)	0.70
< 4000 per mm^3^	83/276 (30.1)	79/262 (30.2)	4 /14 (28.6)	0.67
Lymphocyte count				
Median (IQR) — per mm^3^	1100 (800–1500)	1100 (800–1500)	700 (400–800)	< 0.01
Distribution — no./total no. (%)				
< 1500 per mm^3^	204/276 (75.0)	193/262 (73.7)	11/14 (78.6)	0.72
Eosinocyte count				
Median (IQR) — per mm^3^	0.01 (0–0.03	0.01 (0–0.03)	0 (0–0.01)	0.19
Distribution — no./total no. (%)				
< 100 per mm^3^	126/276 (45.7)	117/262 (44.7)	9/14 (64.3)	0.33
Platelet count				
Median (IQR) — per mm^3^	177,000 (140000–221,000)	177,000 (141000–222,000)	136,000 (112000–196,000)	0.03
Distribution — no./total no. (%)				
< 150,000 per mm^3^	87/276 (31.5)	80/262 (30.5)	7/14 (50.0)	< 0.01
Median hemoglobin (IQR) — g/dl	131.5 (123.0–143.0)	132.0 (124.0–143.0)	130.0 (100.0–139.0)	0.19
Distribution of other findings — no./total no. (%)				
C-reactive protein ≥10 mg/L	162/266 (60.9)	152/252 (60.3)	10/14 (71.4)	0.30
Procalcitonin ≥0.5 ng/ml	6/240 (2.5)	5/226 (2.2)	1/14 (7.1)	0.31
Lactate dehydrogenase ≥250 U/L	31/93 (33.3)	27/88 (30.7)	4/14 (28.6)	0.57
Aspartate aminotransferase > 40 U/L	39/274 (14.2)	34/260 (13.1)	5/14 (35.7)	0.03
Alanine aminotransferase > 40 U/L	61/274 (22.3)	58/260 (22.3)	3/14 (21.4)	0.62
Total bilirubin > 17.1 μmol/L	43/274 (15.7)	42/260 (16.2)	1/14 (7.1)	0.32
Creatine kinase ≥200 U/L	9/81 (11.1)	7/78 (9.0)	2/3 (66.7)	0.03
Creatinine ≥115 μmol/L	6/274 (2.2)	3/260 (1.2)	3/14 (21.4)	< 0.01
D-dimer > 0.5 mg/L	123/229 (53.7)	115/219 (52.5)	8/10 (80.0)	0.08
Myohemoglobin > 80 μg/L	18/204 (8.0)	13/191 (6.8)	5/13 (38.5)	< 0.01
Erythrocyte sedimentation rate > 20 mm/h	180/201 (90.0)	169/190 (88.9)	11/ 11 (100.0)	0.29

^a^ Lymphocytopenia was defined as a lymphocyte count less than 1500 per cubic millimeter. Eosinopenia was defined as an eosinocyte count of less than 100 per cubic millimeter. Thrombocytopenia was defined as a platelet count of less than 150,000 per cubic millimeter. These are results of the first examination after admission

According to the results of the first examination after admission, 30.1% (83/276), 75.0% (204/276), and 31.5% (87/276) of the patients had leukopenia, lymphocytopenia, and thrombocytopenia, respectively. Lymphocytopenia and thrombocytopenia were more obvious in the severe group compared to that in the non-severe group. The C-reactive protein levels were elevated in 60.9% (162/266) of the patients; the erythrocyte sedimentation rate of 90.0% (180/201) of patients and D-dimer levels of 53.7% (123/229) of patients were also elevated. Elevated procalcitonin, creatine kinase, alanine aminotransferase, aspartate aminotransferase, and myoglobin levels were observed in 2.5, 11.1, 14.2, 22.3, and 8.0% of patients, respectively.

### Clinical outcomes

As shown in Table 3, most of the patients (94.9%, 262/276) were cured and discharged from the hospital. Ten out of 276 (3.6%) patients, all of whom belonged to the severe group, showed condition deterioration to a critical status and were transferred to Suizhou Central Hospital, a superior hospital. Eventually, five of them died and five survived. All five people who died received endotracheal intubation; one of the five survivors received endotracheal intubation, which was removed 20 days later. The remaining four only received non-invasive ventilator treatment; 1.4% (4/276) of the patients were transferred to Suizhou Central Hospital for non-COVID-19 reasons and were shortly discharged from the hospital. The median lengths of COVID-19 treatment and hospital stay were 14.0 days (IQR, 11.0–18.0 days) and 18.0 days (IQR, 15.0–24.0 days), respectively.

**Table 3:** Clinical outcomes of patients with COVID-19

Variable	All Patients	Disease Severity		*p* value
	(N = 276)	Non-severe (N = 262)	Severe (N = 14)	
Clinical outcomes — no. (%)				
Cured and discharged from hospital	262/276 (94.9)	258/262 (98.5)	4/14 (28.6)	< 0.01
Deteriorated to critical and transferred to a hospital	10/276 (3.6)	0/262 (0)	10/14 (71.4)	< 0.01
Transferred to a hospital for other reasons	4/276 (1.4)	4/262 (1.5)	0/14 (0)	0.81
Median length of treatment for COVID-19 (IQR) — days ^a^	14.0 (11.0–18.0)	14.0 (11.0–18.0)	16.0 (15.0–17.0)	0.15
Median length of hospitalization (IQR) — days ^a^	18.0 (15.0–24.0)	18.0 (15.0–24.0)	21.5 (14.0–21.0)	0.44

^a^ 10 patients who deteriorated to critical status and 4 patients who were transferred to other hospital for other reasons were not included

## Discussion

Understanding the clinical characteristics of COVID-19 inpatients in the areas around the core COVID-19 outbreak region is very important for controlling the spread of COVID-19 and decision-making for epidemic control. Our study on 276 inpatients in Zengdu Hhospital confirms that COVID-19 patients in the areas surrounding the core COVID-19 outbreak region showed mainly mild and moderate illness with fever and lymphocytopenia as the main clinical features. Older patients (age > 60 years) or those with underlying comorbidities are at higher risk of deteriorating to critical status. The length of hospital-stay and antiviral treatment duration for COVID-19 were slightly longer than those in Wuhan.

All patients who tested positive for COVID-19 by RT-PCR in the study region were admitted to the hospital, regardless of the severity of the patients’ condition. There were several reasons why our hospital established such an admission standard. First, at that time, the outbreak was still in the early stage. The understanding of the epidemic situation in Zengdu District, a residential area approximately 3 h away from the core COVID-19 outbreak region, was very limited, and there was no clear evidence to determine which patients could be treated at home. Second, the representative area of Zengdu District was a key area to control the spread of the epidemic; therefore, it was necessary to treat as many diagnosed patients as possible. Third, at that time, Zengdu Hhospital was supported by 98 Jiangxi volunteer doctors (including most of the authors of this article) and extensive medical equipment. Hence, there were sufficient medical resources to treat all the diagnosed patients. Our admission criteria were formulated under such special circumstances, although this admission standard was different from the current international standard. Moreover, compared to studies in which only seriously ill COVID-19 patients were admitted, our admission criteria better reflect the disease characteristics in the area around the outbreak point, so as to provide a decision-making reference for hospitals in the residential area to decide which patients should stay at home for observation and which high-risk patients should be hospitalized in a timely manner.

The patients in Zengdu area show mainly showed mild and moderate illness, with a few patients showing severe and critical illness. In Wuhan, as the site with the most serious COVID-19 infection in China, many patients did not get timely diagnosis and treatment initially, and medical resources were insufficient to accommodate the sudden burst of patients. As a result, the proportion of severe cases reached 15.0–30.0% [14, 15], while the rate of severe disease in other regions was 3–10% [16, 17], similar to 5.1% in this study. This may be because, with the deepening of the understanding of COVID-19 and the formulation of relevant guidelines [18, 19], many patients were diagnosed and treated in a timely manner without deteriorating into severe disease. Besides, the difference in admission criteria was also a reason why the rate of severe disease in this study was significantly lower than that in Wuhan or abroad.

The early common symptoms of COVID-19 patients include fever, cough, sputum, and other symptoms of lower respiratory tract infection. As the most common symptom, in general, more than 80% of patients have a fever, but only 38.4% of the patients had a fever at the time of admission, which shows that the fever in many patients was intermittent. It also means a large number of patients with intermittent fever will be set free if instant body temperature readings are the only measure used for screening [2, 20]. The proportion of fever in critically ill patients increases significantly after hospitalization, and most of these new fever cases may be caused by secondary infection, so it is necessary for severe patients to receive antibiotics to prevent secondary infection [13].

COVID-19 patients over 60 years old were more likely to show deterioration into critical illness. Previous studies on severe acute respiratory syndrome (SARS) and Middle East Respiratory Syndrome (MERS) have confirmed that age was an important predictor of poor prognosis [20, 21], and similar conclusions were obtained for COVID-19 [22]. Data obtained by Nanshan Zhong et al. [23] and Zhongliang Wang et al. [14] showed that the age of severe patients was significantly older than that of non-severe patients. Consistent with these findings, among the patients we collected, the median age of severe patients was 65 years, while that of non-severe patients was 50 years. In addition, about 78.6% of the severe patients were more than 60 years old. These studies have shown that older COVID-19 patients have a poor prognosis.

COVID-19 patients with comorbidities were also likely to show deterioration [24]. The studies by Nanshan Zhong et al. and DaweiWang et al. [22] both showed high proportions of comorbidities in severe patients. A WHO survey reported that people with comorbidities had a higher risk of severe disease [25]. In a recent retrospective study of 25 death cases with COVID-19 [24], all of the deceased patients have comorbidities, which were considered to be one of the most important risk factors for death. In this study, 85.7% of the severe patients had comorbidities. This may be due to abnormal immune function and increased susceptibility to SARS-CoV-2 in patients with comorbidities [26, 27]. In addition, COVID-19 damage to the lungs can aggravate some comorbidities, such as chronic obstructive pulmonary disease. Antiviral drugs and glucocorticoids also have limited benefits for patients with comorbidities.

In terms of laboratory tests, 75% of patients had lymphopenia, and more obvious findings were noted in severe patients. The novel coronavirus can induce a cytokine storm and inhibit the generation of lymphocytes [28, 29], so lymphopenia is very common in patients with COVID-19. The low absolute value of lymphocytes can be used as a reference indicator for clinical diagnosis of novel coronavirus infections [6]. Lymphocytes showed a pronounced decline in severe patients than in non-severe patients, indicating that the degree of lymphocyte decline can be used to assess the severity of the disease [30], and that continuous decline of lymphocytes is also one of the indicators of disease deterioration [13]. In the absence of nucleic acid detection and CT, this can be an important tool for determining the severity of the disease. The length of hospital stay in this study was slightly longer than that in Wuhan, which was 11–12 days [23, 31]. This contradicted the finding that the length of hospitalization is positively related to disease severity because COVID-19 severity in this study was significantly lower than that in Wuhan. However, the allocation of medical resources is also an important factor affecting the length of hospitalization. The number of infected patients in Wuhan was large and medical resources were scarce, so the hospital had to discharge inpatients as soon as possible to treat newly admitted patients. The inpatient data collected in this study were from a community hospital that was supported by many Jiangxi doctor volunteers and medical supplies, which ensured sufficient medical resources. The characteristics of inpatients under this special medical setup were different from those at other hospitals. In particular, after the local epidemic is mostly controlled, some wastage of medical resources may be inevitable. For example, patients were allowed to stay in the hospital for some time to recover even after meeting the discharge criteria for COVID-19, which was not possible in the hospital in Wuhan. This was also the reason why the length of treatment for COVID-19 (14 days) is significantly shorter than the length of hospitalization (18 days). In addition, hospitals in Wuhan only accept patients who have been diagnosed as showing COVID-19, while hospitals outside Wuhan admitted many patients who were not diagnosed at admission and were also hospitalized for the 1–3 days it took for nucleic acid test results to arrive. Three studies from regions with sufficient medical resources [32–34], namely Taizhou, Guangdong, and Shenzhen, can support our hypotheses since their median hospital stays were 18 days, 20 days, and 20 days, respectively, which were close to the results of this study.

This study has several limitations. First, since it is a retrospective study with a limited number of patients, some conclusions need to be verified by studies with more rigorous design and larger samples. Second, Zengdu Hhospital was a community hospital, and most of the critically ill patients had to be transferred to superior hospitals for treatment. we are temporarily unable to get information on the follow-up treatment and complications of these patients. Third, when calculating the incubation period, we excluded the unclear contact date, resulting in fewer patients included, and the potential memory bias will also affect our results. Fourth, our admission criteria were different from the current internationally recognized criteria, which limits comparability with other studies. However, our admission criteria were set in high-risk areas at the early stage of the epidemic to avoid the spread of the epidemic, which was essential and important. In addition, only PCR-confirmed COVID-19 patients were included in this study and asymptomatic infections without PCR confirmation were omitted, so the characteristics we described are only suitable for PCR-confirmed COVID-19 patients.

## Conclusions

Most of the COVID-19 patients in Zengdu area had mild disease. Older patients with underlying comorbidities had a high risk of progressing to severe disease. A large number of patients with intermittent fever will be omitted by the temperature checks that are currently widely being deployed. The length of hospitalization and antiviral treatment for COVID-19 were slightly longer than those in the Wuhan area. This work will contribute to our understanding of the disease characteristics in the areas around the COVID-19 core outbreak point and provide reference data for decision-making for epidemic prevention and control in such special areas.

## Data Availability

The datasets used and/or analyzed during the current study are available from the corresponding author on reasonable request.

## Abbreviations

COVID-19: Coronavirus disease-19
SARS-CoV-2: Severe acute respiratory syndrome coronavirus 2
CDC: Centers for Disease Control and Prevention
WHO: World Health Organization
RT-PCR: Real-time reverse-transcriptase–polymerase chain reaction
CT: Computed tomography
BMI: Median body mass index
IQR: Interquartile ranges
SARS: Severe acute respiratory syndrome
MERS: Middle East Respiratory Syndrome
ACE: Angiotensin-converting enzyme
